# Chronic Pancreatitis Associated with Chylous Ascites Simulating Liver Cirrhosis

**DOI:** 10.1155/2013/763561

**Published:** 2013-12-02

**Authors:** Wellington Andraus, Lucas Souto Nacif, Raphael L. C. Araujo, Yuri dos Santos Buscariolli, Mayara Salvato, Luiz Augusto Carneiro D'Albuquerque

**Affiliations:** Liver and Gastrointestinal Transplant Division, Department of Gastroenterology, University of São Paulo School of Medicine, 05403-900 São Paulo, SP, Brazil

## Abstract

*Purpose*. Ascites, esophageal varicose veins, and acute digestive bleeding are unusual in the clinical presentation of chronic pancreatitis; however, these symptoms are frequently observed in patients with liver cirrhosis. Moreover, it is unlikely to observe chylous ascites in both presentations. *Method*. We report a patient who presented with chronic pancreatitis with splenic vein thrombosis, necrosis of the pancreatic neck and tail, esophageal varicose veins with previous bleeding, and chylous ascites. After partial pancreatectomy, his treatment was based on low-fat oral diet with medium-chain triglycerides with remarkable resolution of the chylous ascites. After 3 years, he presented with decompensated chronic pancreatitis and underwent plexus alcoholization and biliary-enteric deviation with an unremarkable postoperative course. *Conclusion*. Ascites is rarely associated with chronic pancreatitis, and chylous ascites is even rarer. The treatment of atraumatic chylous ascites is based on resolution of the obstructive causes and should include drainage and a low-fat diet with medium-chain triglycerides.

## 1. Introduction

Liver cirrhosis typically results in hepatocellular failure and portal hypertension, and the clinical presentation varies according to the magnitude of these factors. Chronic liver diseases and cirrhosis together represent the 12th leading cause of death in the United States with a mortality rate of 9.7 per 100.000 inhabitants [1]. Jaundice and ascites are remarkable clinical signs of decompensated cirrhosis, especially if they are associated with a previous diagnosis of esophageal varicose veins and bleeding.

Chronic pancreatitis is a progressive inflammatory disorder in which the pancreatic secretory parenchyma is destroyed and replaced by fibrous tissue, eventually leading to malnutrition and diabetes [2]. The clinical presentation is typically constant and disabling pain with intermittent attacks simulating acute pancreatitis [2]. However, the clinical presentation of ascites in pancreatic diseases is a rare event. Among the types of ascites, the chylous ascites is defined as a pathological accumulation of chylous fluid in the peritoneal cavity. Generally, it is related to an unrecognized intraoperative disruption of major retroperitoneal lymphatic vessels due to surgical trauma. Although the causes of chylous ascites without a history of previous surgery are rare in clinical practice, the presence of malignancy, trauma, cirrhosis, tuberculosis, and less frequently acute pancreatitis should be included among the differential diagnoses [3, 4].

We report a patient who presented with chronic pancreatitis with acute decompensating associated with splenic vein thrombosis, esophageal varicose veins with previous bleeding, and chylous ascites caused by chronic pancreatitis. It was initially considered as a putative chronic decompensated hepatic dysfunction. To our knowledge, this is the first case describing chronic pancreatitis simulating cirrhosis at the initial presentation.

## 2. Method

The patient was a 37-year-old man with diabetes mellitus non-insulin-dependent. He was a smoker and chronic alcohol drinker. His first clinical evaluation was done in 2008. He presented with weight loss (7 kilos in 6 months), esophageal varicose veins, previous history of upper digestive tract hemorrhage, and progressive increase of abdominal circumference due to ascites. Based on the signs of portal hypertension, he was referred to the liver transplantation service for a putative diagnosis of chronic advanced hepatic disease.

**Figure 1:**
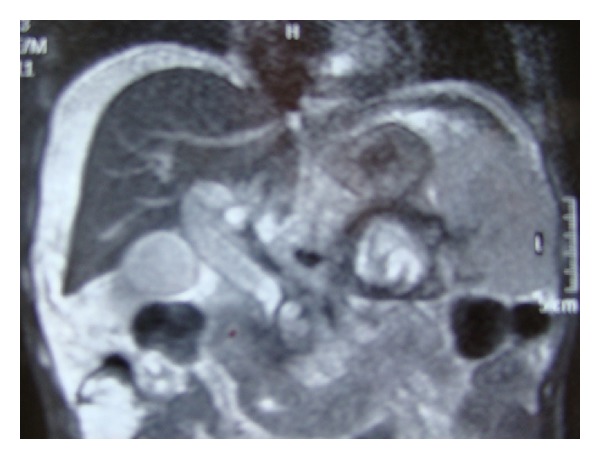
Preoperative magnetic resonance image showing retropancreatic collection, partial necrosis of the pancreatic body, normal liver appearance, and ascites.

The admission laboratory exams did not show irregular liver enzymes. The following paracentesis demonstrated an opaque liquid with elevated level of triglycerides to 12.8 mmol/L (over than 5.2 mmol/L) confirming the presence of the chylous ascites [5]. Computerized tomography demonstrated retropancreatic collections, partial necrosis of the pancreatic tail, and thrombosis of the splenic vein. However, the patient had a normal liver appearance, as also verified at magnetic resonance and demonstrated in Figure 1.

He underwent distal pancreatic necrosectomy (neck and tail) and splenectomy, and 3 liters of chylous ascites was observed in the operation. The spleen had collateral veins to the stomach and left liver making the splenectomy very difficult. No other findings were observed during the surgery. Cavity drainage was performed, and he presented with chylous ascites after surgery that diminished daily and was resolved within 11 days. The postoperative treatment was based on the control of infection process and low-fat oral diet with medium-chain triglycerides. An abdominal computerized tomography was performed after the postoperative antibiotic course (14 days of 3rd generation cephalosporin and metronidazole) and confirmed the ascites resolution. He was discharged on the 16th postoperative day. The endoscopy follow-up performed after discharge also showed a decrease of the esophageal varicose veins.

After 6-month follow-up, he presented no signs or symptoms of decompensating pancreatic function or ascites. However, he resumed alcohol consumption and presented with a recurrence of the disease after 3 years. In 2011, he had a diffuse increase of the residual pancreas (head) coursing with abdominal pain and biliary obstruction. He underwent another surgery when a biliary-enteric anastomosis and celiac plexus alcohol infusion were performed. This postoperative course was also unremarkable, and he was discharged on the 8th postoperative day. His follow-up was four years after the first surgery (10 months after the second surgery). He presented with diabetes non-insulin-dependent and had ceased alcohol consumption after the last surgery.

## 3. Discussion

Chronic pancreatitis simulating cirrhosis with acute abdominal pain, high gastrointestinal bleeding, and ascites is an unexpected association. To our knowledge, this is the first case with this presentation. Pancreatitis-induced splenic vein thrombosis is a disorder that can occur as a sequel to both acute and chronic pancreatitis, varying, respectively, from 12.4% to 22.6% [6]. In a systematic review of atraumatic chylous ascites, cirrhotic liver was considered responsible for 11% of cases, while pancreatitis, both acute and chronic, accounted for only 4% and abdominal distension only 1% [7]. Splenic vein thrombosis is associated with upper digestive tract hemorrhage but is rarely associated with ascites [6]. Considering the presence of chylous ascites as a consequence of chronic pancreatitis with splenic vein thrombosis, it is even more unexpected and was not reported in any recent systematic review [6].

In this case, it was considered that the inflammation of the acute course of chronic pancreatitis, leading to pancreatic necrosis, and splenic vein thrombosis elevated the pressure in the intestinal lymphatic drainage system, resulting in its leakage. Facing this scenario, this patient was referred to our service with the typical signs and symptoms of decompensated liver cirrhosis (ascites, esophageal varicose veins, and previous variceal bleeding). We considered splenic vein thrombosis and pancreatic necrosis only after preoperative imaging and laboratory tests. After he underwent surgery, which included necrosectomy, splenectomy, and drainage of the abdominal cavity, the patient was placed on a low-fat oral diet with medium-chain triglycerides, and the chylous ascites was resolved. As the management of chylous ascites remains controversial, some successful strategies have been reported.

Although previously suggested in the literature, analogs of somatostatin and parenteral nutrition were not used. Our patient had successful evolution using only low-fat diet and this decision did not compromise the postoperative course, in contrast to other reports [8, 9]. We believe that diet should be the first therapeutic option in atraumatic chylous ascites for putative small damages in the intestinal lymphatic system, unlike traumatic chylous ascites, which typically occurs after retroperitoneal surgery. Six months after surgery, he resumed alcohol consumption. Following the clear association between alcohol ingestion and chronic pancreatitis [10], the pancreatic fibrosis process continued to develop in the head, caused obstruction of the common bile duct, and again became painful, demanding surgical intervention. Consequently, a psychological follow-up was required facing his alcohol relapse after six months of abstinence.

In summary, we reported this case in an attempt to highlight a rare association between abdominal pain, ascites, and esophageal varicose veins. These symptoms were all caused by chronic pancreatitis and not liver cirrhosis, which would be more congruent with this description. The decompensated chronic pancreatitis causing atraumatic chylous ascites made this case even more unexpected. However, we suggest the inclusion of chylous ascites as a possible event in patients presenting with pancreatitis-induced splenic vein thrombosis.
